# Pre-emergence herbicides widely used in urban and farmland soils: fate, and potential human and environmental health risks

**DOI:** 10.1007/s10653-024-01907-6

**Published:** 2024-03-14

**Authors:** Aney Parven, Islam Md Meftaul, Kadiyala Venkateswarlu, Saianand Gopalan, Mallavarapu Megharaj

**Affiliations:** 1 Global Centre for Environmental Remediation (GCER), College of Engineering, Science and Environment, The University of Newcastle, ATC Building, University Drive, Callaghan, NSW 2308 Australia; 2 Department of Agricultural Chemistry, Sher-e-Bangla Agricultural University, Dhaka, 1207 Bangladesh; 3 Formerly Department of Microbiology, Sri Krishnadevaraya University, Anantapuramu, 515003 India; 4 crcCARE, University Drive, Callaghan, NSW 2308 Australia

**Keywords:** Weedicides, Urban and agricultural settings, Sorption‒desorption, Leaching potential, Nontarget biota, Ecological risks

## Abstract

We determined the distribution, fate, and health hazards of dimethenamid-P, metazachlor, and pyroxasulfone, the effective pre-emergence herbicides widely used both in urban and agricultural settings globally. The rate-determining phase of sorption kinetics of these herbicides in five soils followed a pseudo-second-order model. Freundlich isotherm model indicated that the herbicides primarily partition into heterogeneous surface sites on clay minerals and organic matter (OM) and diffuse into soil micropores. Principal component analysis revealed that soil OM (*R*^2^, 0.47), sand (*R*^2^, 0.56), and Al oxides (*R*^2^, 0.33) positively correlated with the herbicide distribution coefficient (*K*_d_), whereas clay (*R*^2^, ‒ 0.43), silt (*R*^2^, ‒ 0.51), Fe oxides (*R*^2^, ‒ 0.02), alkaline pH (*R*^2^, ‒ 0.57), and EC (*R*^2^, ‒ 0.03) showed a negative correlation with the *K*_d_ values. Decomposed OM rich in C=O and C–H functional groups enhanced herbicide sorption, while undecomposed/partially-decomposed OM facilitated desorption process. Also, the absence of hysteresis (H, 0.27‒0.88) indicated the enhanced propensity of herbicide desorption in soils. Leachability index (LIX, < 0.02–0.64) and groundwater ubiquity score (GUS, 0.02‒3.59) for the soils suggested low to moderate leaching potential of the herbicides to waterbodies, indicating their impact on water quality, nontarget organisms, and food safety. Hazard quotient and hazard index data for human adults and adolescents suggested that exposure to soils contaminated with herbicides via dermal contact, ingestion, and inhalation poses minimal to no non-carcinogenic risks. These insights can assist farmers in judicious use of herbicides and help the concerned regulatory authorities in monitoring the safety of human and environmental health.

**Graphical abstract:**

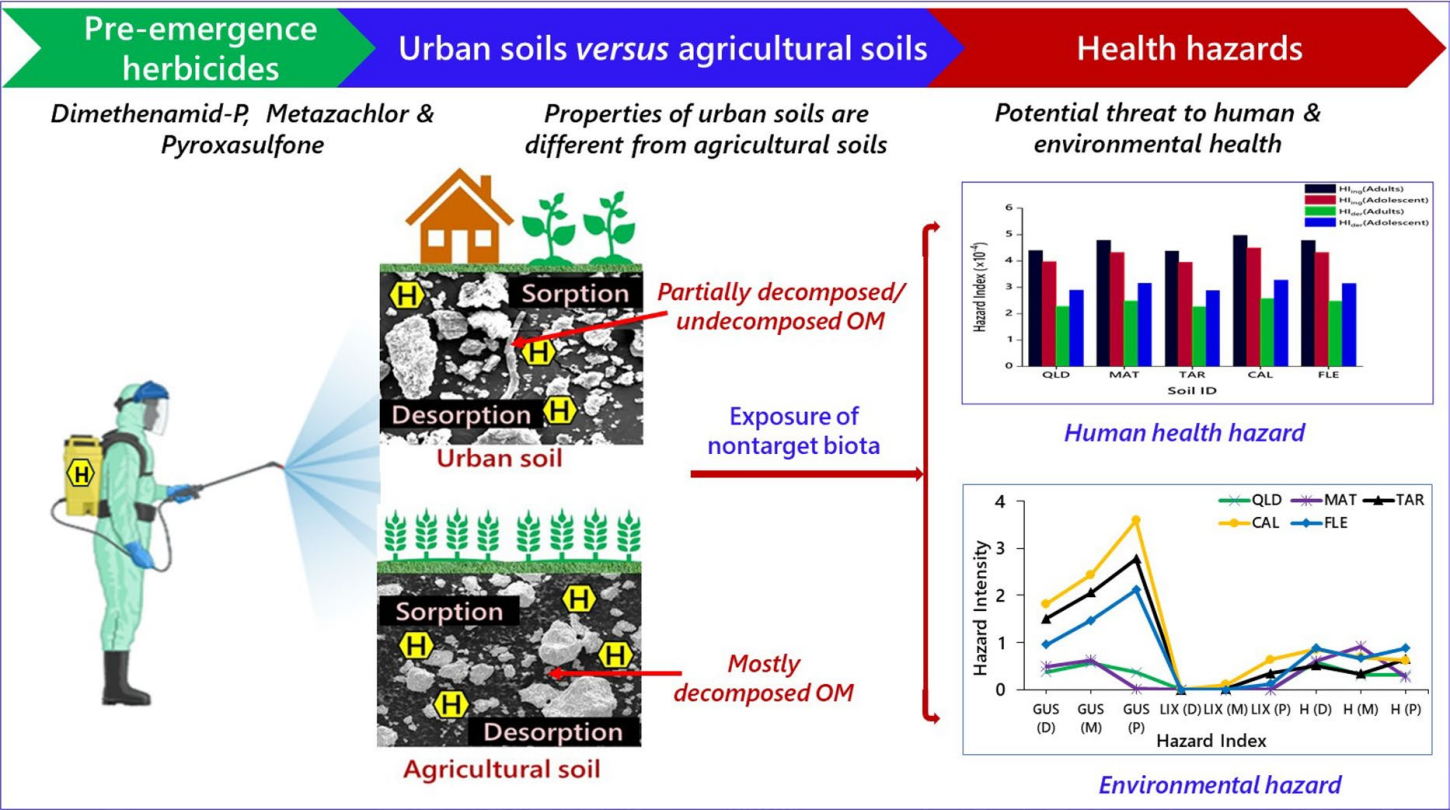

**Supplementary Information:**

The online version contains supplementary material available at 10.1007/s10653-024-01907-6.

## Introduction

Chloroacetamide herbicides of the Group 15 (http://www.wssa.net/Weeds/Resistance/WSSA-Mechanism-of-Action.pdf) are the pre-emergence weedicides that have been used most commonly to control both annual grasses and broad-leaved weeds during cultivation of commercial crops such as cotton, corn, peanut, soybean, sorghum, sunflower, etc. (Westra et al., 2015). Among them, dimethenamid-P (S-2-chloro-N-(2,4-dimethyl-3-thienyl)-N-(2-methoxy-1-methylethyl) acetamide) and metazachlor (2-chloro-N-(2,6-dimethylphenyl)-N-(1H-pyrazol-1-ylmethyl) acetamide) inhibit biosynthesis of long-chain fatty acids implicated in cell division and elongation process (Velisek et al., 2020; Walsh et al., 2015). Pyroxasulfone (3-[5-(difluoromethoxy)-1-methyl-3-(trifluoromethyl)pyrazol-4-ylmethylsulfonyl]-4,5-dihydro-5,5-dimethyl-1,2-oxazole) is a pyrazole-based pre-emergence herbicide with a similar mode of action as chloroacetamides (Tanetani et al., 2009). These novel pre-emergence herbicides thus control weeds that are even resistant to glyphosate (Westra et al., 2015). Nowadays, herbicides have become an inherent constituent of modern farming to considerably increase crop yield by reducing the negative effects of weeds (Dayan, 2019). Without the use of pesticides, global production would be reduced up to 78% of fruits, 54% of vegetables, and 32% of cereals (Tudi et al., 2021). Every year over 4.10 million tons of pesticides have been used worldwide, wherein herbicides contribute, by volume, to around 60% (Dayan, 2019). Only about 1% of the applied herbicide reaches the target weeds, and the remaining bulk amount enters the environment causing detrimental impacts on nontarget biota (Arias-Estévez et al., 2008; Parven et al., 2021). Consequent to the extensive use of the herbicdes, the residues and their metabolites have been often detected in soil, water and, the environment (Kaur et al., 2021).

Although herbicides play a key role in achieving food security for the ever-increasing world population, they are a source of anthropogenic contamination of ecosystems (Velisek et al., 2020). In particular, chloroacetamide herbicides enter the environment and biological systems through soil and water contamination, persist for few months to several years and cause significantly toxic effects to humans and wildlife (Ma et al., 2021; Sousa et al., 2020). The residues of chloroacetamide herbicides in soil affect subsequent rotation crops, mostly in sandy soils with less organic matter (OM) content (Mahanta et al., 2023). Short-term exposure to dimethenamid-P showed an adverse effect on earthworms (*Eisenia andrei*) (Lackmann et al., 2021). Metazachlor was associated with toxicity to aquatic biota like crayfish (*Procambarus virginalis*) (Velisek et al., 2020), macrophytes (Mohr et al., 2008), and phytoplankton communities (Wijewardene et al., 2021), while exposure to pyroxasulfone increased the incidence of urothelial effects and urinary crystal formation in male rats (Kyoya et al., 2020).

The sorption and desorption of herbicides are influenced by the physicochemical properties of the soil, the chemical used, and the microbial activities within the soil (Sarkar et al., 2020). Indeed, soil texture and chemical properties play a vital role in herbicide binding and subsequent weed control (Westra et al., 2015). It has also been demonstrated that other chloroacetamide herbicides like s-metolachlor, acetochlor and alachlor bind to soil components, wherein soil OM plays a predominant role (Westra et al., 2015). However, no comprehensive data are available to gain a more profound understanding of the potential health hazards posed by dimethenamid-P, metazachlor, and pyroxasulfone to both humans and other biota. Here we tested whether the differential physicochemical soil properties of urban and agricultural settings would contribute to the overall fate and effects of these three herbicides. Thus, this study investigated the sorption‒desorption, fate, and movement of the three pre-emergence herbicides, viz., dimethenamid-P, metazachlor, and pyroxasulfone, in two urban and three agricultural soils besides determining their potential risk of exposure toward nontarget biota.

## Materials and methods

### Soil sampling

**Fig. 1:**
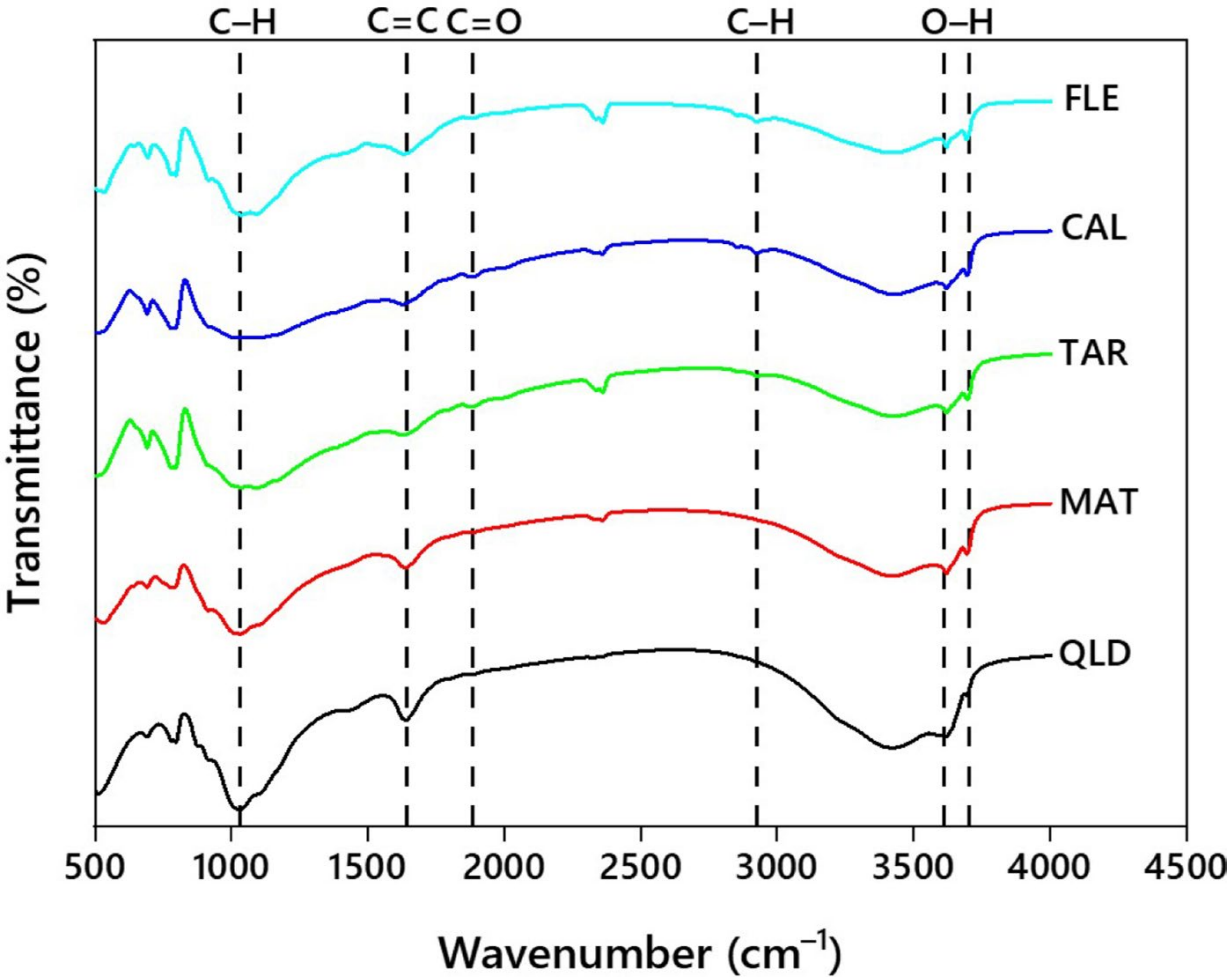
Fourier-transform infrared (FTIR) spectra representing the functional groups of organic matter present in urban soils CAL and FLE, and agricultural soils QLD, MAT, and TAR

**Fig. 2:**
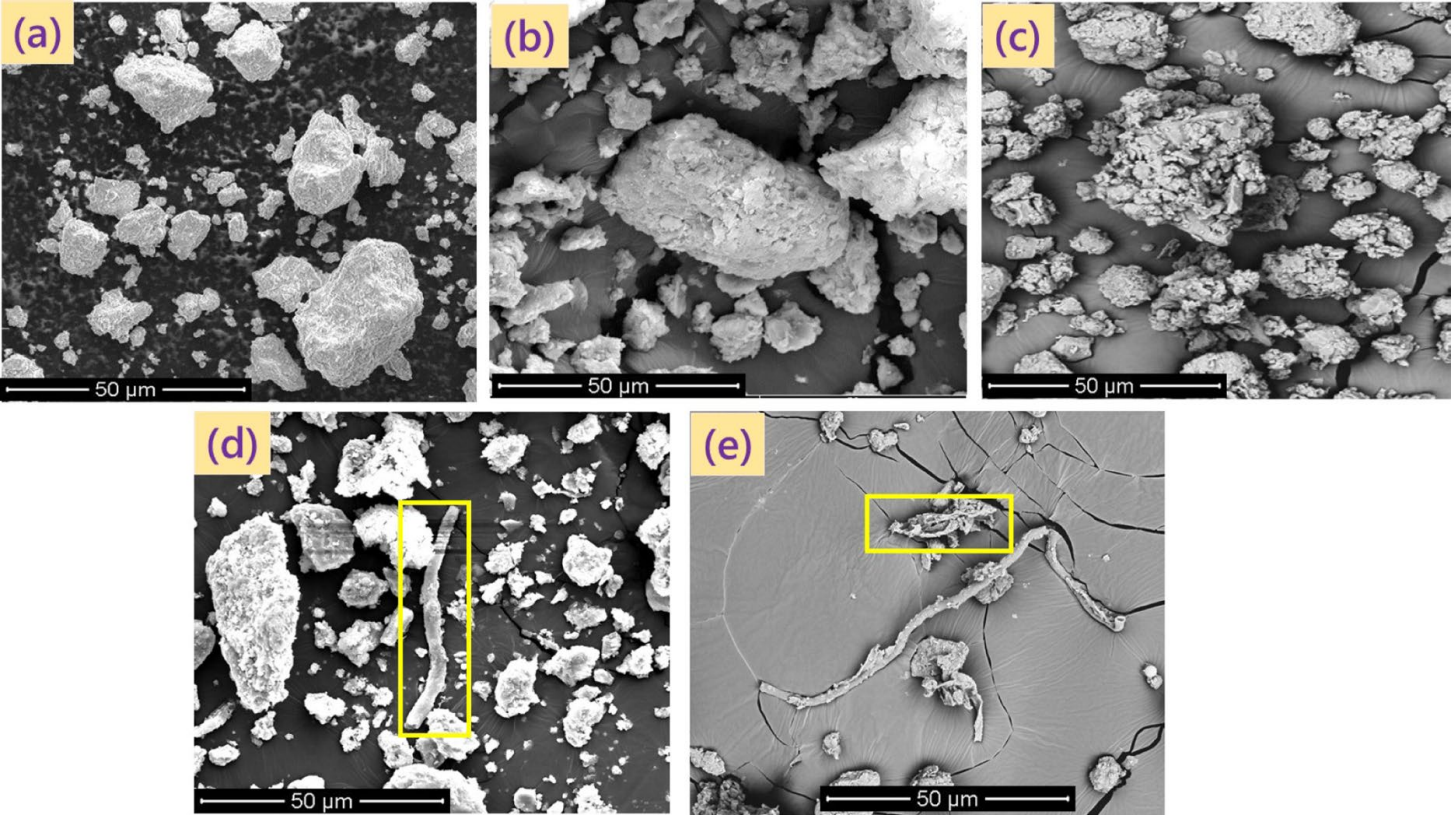
SEM image of agricultural soils **a** QLD, **b** MAT, and **c** TAR. Urban soils **d** CAL and **e** FLE showing undecomposed and partially decomposed OM

**Fig. 3:**
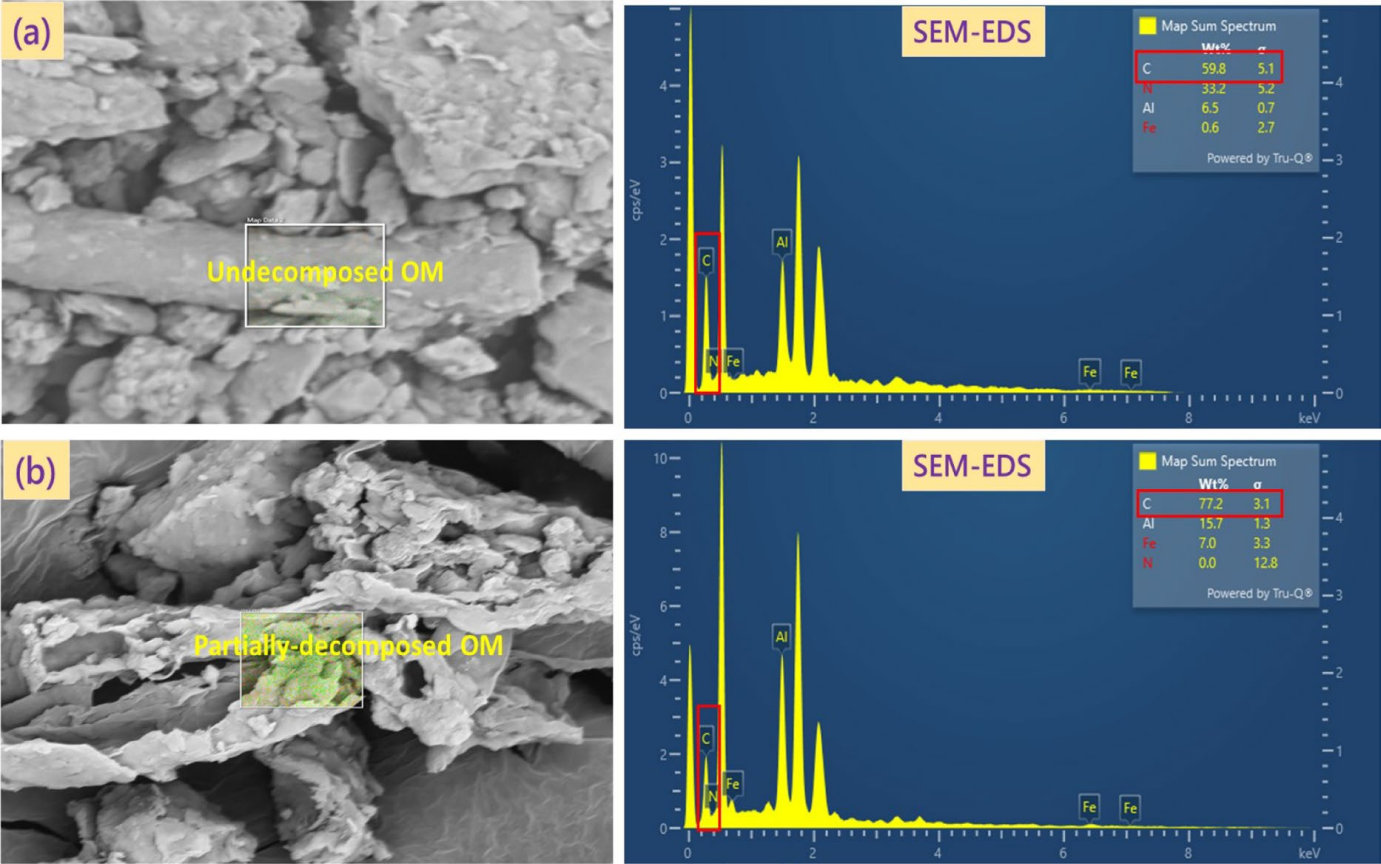
SEM–EDS of the corresponding C-loaded OM in urban soils **a** CAL, and **b** FLE, highlighting C on per cent weight basis, and their relative molecular positions (right panels)

Two urban and three agricultural soils from different locations, involved in growing crops, were collected at 0–20 cm depth from soil surface. Composite samples were prepared by thoroughly mixing bulk samples (*n* = *5*) that were randomly collected from each selected location. The soil samples were air-dried, sieved through a 2-mm dia mesh, and subsequently stored at a temperature of 21 ± 1 °C until further use. Soil textural classification, pH and EC, total organic carbon (TOC), functional groups of soil OM (Fig. 1; Table S2), oxides of Al and Fe, and minerals were determined (Table S1) following the standard methods described earlier by Meftaul et al. (2021). Both morphology (Fig. 2) and surface elemental composition of OM (Fig. 3) in the soils were assessed using scanning electron microscope (SEM) and energy-dispersive spectroscopy (EDS) (Bruker Nano GmbH, Germany). EDS elemental mapping was obtained at 12 keV with 10-μm step intervals and an integration time of 0.50 s/step.

### Chemicals

Dimethenamid-P (≥ 98%), metazachlor (≥ 98%), and pyroxasulfone (≥ 95%) were obtained from Merck. The commercial formulations, Outlook (dimethenamid-P, 720 g/L), Butisan (metazachlor, 500 g/L), and Sakura (pyroxasulfone, 850 g/kg), that have been recently used in Australian agriculture, were procured from CRT Raymond’s Warehouse, Australia. Stock solutions of 100 mg/L were prepared from analytical grade dimethenamid-P, metazachlor, and pyroxasulfone in aqueous solution. Calibration curves were developed for three herbicides using multiple working solutions (0.00195–1.0 mg/L) along with blanks of the standards and commercial formulations. For batch sorption‒desorption experiments, aliquots of the commercial formulations, viz., 138.80 μL of Outlook, 200 μL of Butisan, and 117.60 μg of Sakura, were diluted to 1.0 L with Milli-Q water to obtain 100 mg/L stock solutions. Subsequently, the stock solutions were diluted to obtain the working concentrations of 0.50, 1.0, 2.0, 3.0, and 4.0 mg/L.

### Sorption‒desorption studies

Batch experiments of sorption‒desorption were conducted following OECD serial method (OECD, 2000) to obtain sorption and desorption isotherms and kinetics of dimethenamid-P, metazachlor, and pyroxasulfone using the selected urban and agricultural soils. To ensure accuracy of the data, each experiment was carried out using triplicate samples.

### Chemical analysis

The concentrated extracts of dimethenamid-P, metazachlor, and pyroxasulfone were analyzed in an LC–MS using a Zorbax Eclipse plus C18 column (4.60 × 150 mm, 3.50 μm dia) from Agilent Technologies, USA. Column temperature was set at 50 °C for dimethenamid-P and metazachlor, and 40 °C for pyroxasulfone throughout the analysis. The mobile phase used for dimethenamid-P and metazachlor was aqueous 0.0125% acetic acid solution (A) and acetonitrile (B) @ 0.40 mL/min flow rate. The gradient that started with 60% B at 0.0 → 1.0 min was linearly ramped to 100% B at 6 → 11 min followed by a run with 30% B at 12 min and a 6 min post-run (Chen et al., 2018; Lazartigues et al., 2011). For pyroxasulfone, the binary mobile phase was 1% aqueous acetic acid (A) and acetonitrile (B) with a flow rate set at 0.50 mL/min and a gradient started with 10% B at 0 → 0.50 min and raised to 50% B at 15 → 20 min, 100% B at 25 → 30 min and 10% B at 31 min with a post-run time of 1.0 min. The residue analysis was performed in a positive mode following selective ion monitoring for dimethenamid-P (168 → 276 amu) and metazachlor (210 → 278 amu), and a negative mode for pyroxasulfone (179 → 390 amu). Other instrumental parameters followed were: 35 psi nebulizer pressure, 12 mL/min drying gas flow at 300 °C, 3 mL/min sheath gas flow at 150 °C, fragmentor voltage of 100 V, and capillary voltage of 4000 V. Following the above methods, the standard curves developed were linear for dimethenamid-P, metazachlor, and pyroxasulfone with *R*^2^ values of 0.9832, 0.9933, and 0.9945, respectively. The values of limit of detection (LOD) and limit of quantitation (LOQ) were in the range of 0.00097–0.00195 mg/L for the three herbicides. The range in mean recovery (*n* = 3) for the spiked herbicides was 0.00195–1.0 mg/L. The per cent recoveries for dimethenamid-P, metazachlor, and pyroxasulfone were in the range of 81–122, 83–119, and 90–113, respectively, indicating the precision and reliability of the method for herbicide residue analysis in the soil matrix. Agilent OpenLAB CDS ChemStation software was used to process the data.

### Kinetic models

To comprehend the uptake and release rates of herbicide molecules in the soil aqueous phase, kinetic models, the following equations were used for data analysis (Ozbay et al., 2018):

1 $$\log \left( {Q_{max} - Q_{t} } \right) = \log Q_{max} - K_{1} t$$

2 $$\frac{t}{{Q_{t} }} = \frac{1}{{Q_{max}^{2} K_{2} }} + \frac{1}{{Q_{max} }}t$$

where *Q*_*t*_ (mg/kg) represents the extent of sorbed molecules at time ‘t’, and *K*_1_ and *K*_2_ (dimensionless) are the rate constants for pseudo-first-order and pseudo-second-order kinetics, respectively.

### Isotherm models

To gain insights into the sorption–desorption processes of dimethenamid-P, metazachlor, and pyroxasulfone in soil aqueous phase, Langmuir and Freundlich models were employed. The Langmuir isotherm implies monolayer sorption at homogeneous surface sites, where all sorption sites exhibit equal affinity to sorbed molecules and do not interact with each other. The linear equation used to describe this isotherm is as follows (Özbay et al., 2015; Ozbay et al., 2018):

3 $$\frac{{C_{e} }}{{Q_{e} }} = \frac{{C_{e} }}{{q_{max} }} + \frac{1}{{Q_{max} K_{L} }}$$

where C_e_ (mg/L), Q_max_ (mg/kg), Q_e_ (mg/kg) and *K*_L_ (L/mg) denote the solution concentration at the equilibrium, sorption capacity, sorbed concentrations at the equilibrium, and Langmuir constant, respectively. The Freundlich model that relies on the following widely accepted equation, suggests multilayer sorption onto heterogeneous surface sites:

4 $$\log Q_{e} = \frac{1}{n}\log C_{e} + \log K_{F}$$

where 1*/n* (dimensionless) represents the sorption intensity, indicating the favorability of sorption, and *K*_F_ (mg^1−1/n^ L^1/n^/g) is the Freundlich constant, depicting the sorptive attraction of herbicide molecules in the soil matrix. The hysteresis index (H) is indicative of a delay or lag in the desorption process and is determined using the following equation (Meftaul et al., 2023):

5 $$H = \left( {1/n_{des} } \right)/\left( {1/n_{sor} } \right)$$

where 1/n is the Freundlich exponent associated with desorption and sorption isotherms.

### Human health risk assessment

Human health risk assessment was conducted using equations recommended by the US EPA (2020). Exposure pathways, including ingestion, dermal contact, and inhalation to herbicide-contaminated soil, were considered for assessing health risks in adults and adolescents. The non-dietary chronic daily intake (CDI), expressed as mg/kg/day, through these pathways was determined using the following equations (Bhandari et al., 2020):

6 $${\text{CDI}}_{{{\text{ing}}}} = \frac{{C_{{{\text{soil}}}} \times EF \times ED \times IR_{ing} }}{AT \times BW} \times CF$$

7 $${\text{CDI}}_{{{\text{der}}}} = \frac{{C_{{{\text{soil}} }} \times SA \times AF \times SAF \times EF \times ED}}{AT \times BW} \times CF$$

8 $${\text{CDI}}_{{{\text{inh}}}} = \frac{{C_{{{\text{soil}} }} \times EF \times ED \times IR_{inh} }}{PEF \times AT \times BW}$$

In this context, CDI denotes the projected chronic daily intake resulting from the ingestion (CDI_ing_), dermal contact (CDI_der_), and inhalation (CDI_inh_) of soil particles contaminated with herbicide. C_soil_ (mg/kg) is the concentration of the herbicide in the contaminated soil, ED is the duration (in years) of exposure, EF (days/year) is the frequency of exposure, IR_ing_ (mg/day) is the soil ingestion rate, BW (kg) is the body weight of the exposed population, AT is the average lifetime (in days), CF (kg/mg) is the conversion factor, AF (mg/cm) is the skin adherence factor of soil, SA (cm^2^/day) refers to the exposed skin area, SAF (dimensionless) is the skin absorption factor, PEF (m^3^/kg) is the particle emission factor, and IR_inh_ (m^3^/day) is the inhalation rate. These constant parameters and their values available in the literature (APVMA, 2022; Bhandari et al., 2020), used for estimating carcinogenic and non-carcinogenic risks of the selected herbicides in adult and adolescent humans, are presented in Table 1.

**Table 1:** Constant parameters and their values for the estimation of carcinogenic and non-carcinogenic risk of the selected herbicides in humans (based on Bhandari et al., 2020; Meftaul et al., 2023)

S. No	Exposure factor	Adult	Adolescent
1	Ingestion rate (IR_ing_)	100 mg day^−1^	100 mg day^−1^
2	Body weight (BW)	62 kg	32 kg
3	Averaging lifetime (AT)	70 yrs (25,550 days)	70 yrs (25,550 days)
4	Exposed skin area (SA)	5,700 cm^2^ day^−1^	2,800 cm^2^ day^−1^
5	Exposure duration (ED)	30 yrs	14 yrs
6	Exposure frequency (EF)	350 days yr^−1^	350 days yr^−1^
7	Skin adherence factor (AF)	0.07 mg cm^−2^	0.2 mg cm^−2^
8	Skin absorption factor (SAF)	0.13 mg cm^−2^	0.13 mg cm^−2^
9	Conversion factor (CF)	1 × 10^−6^ kg mg^−1^	1 × 10^−6^ kg mg^−1^
10	Carcinogenicity slope factor (SF)	6.20 × 10^−4^ mg kg^−1^ day^−1^	6.20 × 10^−4^ mg kg^−1^ day^−1^
11	Inhalation rate (IR_ih_)	17.50 m^3^ day^−1^	17.50 m^3^ day^−1^
12	Particle emission factor (PEF)	1.36 × 10^9^ m^3^ kg^−1^	1.36 × 10^9^ m^3^ kg^−1^

Maximum acceptable oral doses (Reference doses, RfDs) in adults and adolescents for dimethenamid-P, metazachlor, and pyroxasulfone are 0.03, 0.20 and 0.02 mg/kg/day, respectively as per APVMA (2022)

The non-cancer risks (NCR) associated with dimethenamid-P, metazachlor, and pyroxasulfone exposure were determined using their hazard quotient (HQ). The hazard index (HI), which represents the cumulative HQ of each herbicide, is computed using the following equations (Bhandari et al., 2020; US EPA, 2020):

9 $$HQ = \frac{CDI}{{RfD}}$$

10 $$HI = \sum HQ_{Herbicides}$$

where RfD is the reference dose of a herbicide expressed as mg/kg/day.

### Environmental health risk assessment

The environmental health hazards of the selected herbicides were assessed using groundwater ubiquity score (GUS), and leachability index (LIX) (Martins et al., 2018; Meftaul et al., 2023). The GUS and LIX values were determined based on the *K*_*oc*_ (L/kg) value and half-life (*t*_1/2_, in days) of the herbicide in soil:

11 $${\text{GUS}} = \log {\text{t}}^{1/2} \left( {4 - \log Koc} \right)$$

12 $$LIX = \exp \left( { - k \times Koc} \right)$$

In this context, *k* (per day) represents the first-order rate constant of the herbicides.

13 $$Koc = \frac{{K_{d} }}{\% OC} \times 100$$

14 $$K_{d} = \frac{{Q_{e} }}{{C_{e} }}$$

where %OC represents the proportion of soil organic carbon, and *K*_d_ denotes the distribution coefficient derived from the isotherm range at a specific concentration.

### Statistical analysis

The experimental data obtained were analysed using Microsoft Excel-2016. Linear fitting of isotherms and kinetic models, as well as principal component analysis (PCA), were conducted using OriginProLab2022 software. PCA was done to assess the correlation between the distribution coefficient (*K*_d_) of the herbicides and properties of the selected urban and agricultural soils.

## Results and discussion

### Sorption kinetics of the selected herbicides

Sorption kinetics of dimethenamid-P, metazachlor, and pyroxasulfone sorption were done for describing their fate and behaviour in the chosen urban and agricultural soils and the data obtained are presented in Figs S1 and S2, and Table S3. The results revealed that the urban and agricultural soils rapidly sorbed the herbicides from the aqueous phase at the beginning, followed by a rapid decline and a slow sorption to attain a steady state, finally reached an equilibrium phase at 24 h. Such an initial increased sorption of the herbicides might be because of the fairly abundant and readily available active sorption sites onto the surfaces of soil OM and clay minerals, while the subsequent slow sorption could be due to the gradual diffusion of the herbicides into soil micropores or cross-linked regions of OM (Blachnio et al., 2023). In fact, the per cent sorption of dimethenamid-P, metazachlor, and pyroxasulfone at the equilibrium phase for all the five soils was in the range of 68‒83, 65‒76, and 58‒79, respectively. To begin with, the surface sorption was dominant, and was followed by a gradual diffusion into soil micropores resulting in an increased retention time. Consequently, there was an intra-particle diffusion resulting in a declined sorption before attaining an equilibrium state (Ozbay et al., 2018), suggesting that the herbicide sorption was enhanced in soil aqueous phase due to the accessibility of surface sorption sites rather than herbicide concentrations. Sorption kinetics of dimethenamid-P, metazachlor, and pyroxasulfone in urban and agricultural soils were fitted using the pseudo-first-order kinetic model with lower *R*^2^ (*P* < 0.05) that were in the range of 0.68–0.97, 0.80–0.95, and 0.82–0.93 for dimethanamid-P, metazachlor, and pyroxasulfone, respectively (Fig. S1; Table S3). In contrast, the sorption kinetic data of the herbicides fitted well using pseudo-second-order model for all the five soils as evident from consistent higher values of *R*^2^ (0.99–1.0,* P* < 0.05) (Fig. S2; Table S3).

In the present study, the per cent herbicide sorption in the soils followed the order: QLD < MAT < TAR < FLE < CAL, and was directly proportional to soil OM, in terms of total organic carbon (TOC). Both the urban soils that contained higher amounts of TOC sorbed slightly higher amounts of herbicides than those collected from the farmlands. For instance, the per cent sorption of dimethenamid-P, metazachlor, and pyroxasulfone was maximum in urban soil CAL was 83, 76 and 79, respectively, and the corresponding values for soil FLE were 82, 76 and 76, respectively as these soils contained higher amounts of TOC, while the herbicide sorption was minimum (71, 70 and 75%) in soil QLD since TOC content was only 0.19% (Table S1). However, soil QLD that contained the highest amount of clay (30%) and only 0.19% TOC, sorbed 71, 70 and 75% of dimethenamid-P, metazachlor, and pyroxasulfone, respectively. Soil TAR with 55% silt and 2.02% TOC sorbed the herbicides higher than QLD and MAT, indicating that soil TOC as well as silt and clay minerals play a significant role in herbicide sorption in both urban and agricultural soils. Of the two urban soils, soil CAL contained significantly higher amounts of TOC (7.66%) than FLE (1.29%) (Table S1). However, there was a little difference in herbicide sorption among these soils could be ascribed to the occurrence of either undecomposed or partially-decomposed OM that was seen floating in the centrifuge tubes, which could also be confirmed by SEM and SEM–EDS images (Figs. 2 and 3). In particular, soil CAL contained a greater amount of TOC (7.66%) and was mostly in undecomposed form, whereas TOC in soil FLE (1.29%) was in partially-decomposed state (Fig. 3), indicating the role of both the quantity and quality of soil OM in overall herbicide sorption (Ren et al., 2018). Indeed, decomposed OM/kerogen rapidly enhances sorption and decreases desorption of herbicides (Ozbay et al., 2018). Though soil TAR had higher amounts of TOC (2.02% in decomposed form) and silt (55%) than in soil FLE (1.29% TOC, and 23.8% silt), it sorbed lesser amounts of herbicide, which can be ascribed to slightly alkaline pH (7.51). Likewise, soil QLD that was alkaline with a pH of 9.15 sorbed the least quantity of herbicide among all the five soils, indicating that soils with alkaline pH were less conducive to herbicide sorption than acidic pH. Quartz, being a predominant mineral constituent in all the selected urban and farmland soils, exerted a positive influence on herbicide sorption, particularly in those soils that contained TOC < 1.0%, and this finding is in conformity with those reported earlier by Cheng et al. (2012) and Ren et al. (2018).

### Sorption isotherms for the selected herbicides

**Fig. 4:**
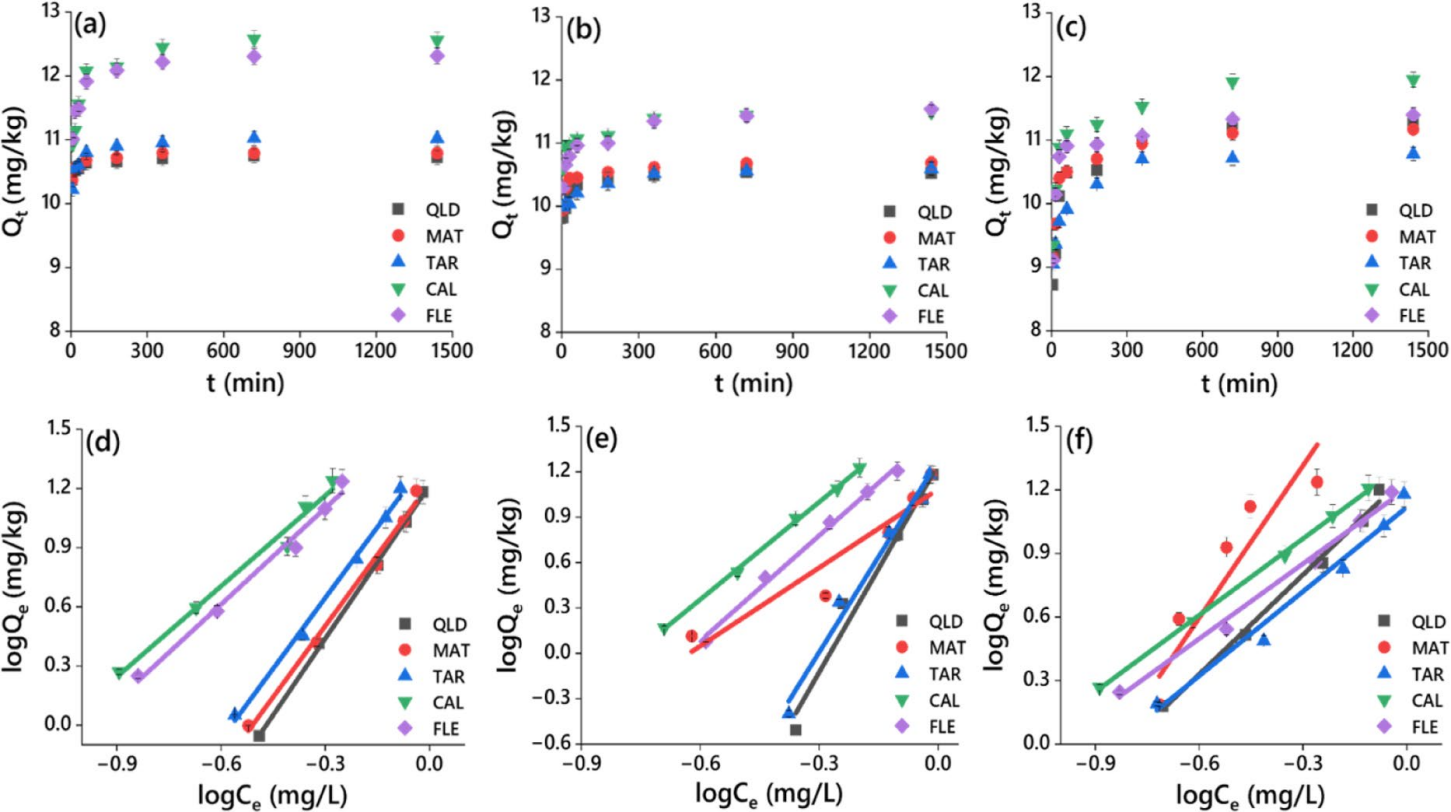
Equilibrium curves **a**, **b**, **c**, and Freundlich isotherms **d**, **e**, **f** of dimethenamid-P, metazachlor, and pyroxasulfone sorption in urban soils CAL and FLE, and agricultural soils QLD, MAT, and TAR. Q_t_ (mg/kg) is the amount of sorbed herbicide at time ‘t’; C_e_ (mg/L) is the equilibrium concentration and Q_e_ (mg/kg) is the equilibrium sorbed concentration of the herbicide

The results presented in Fig. 4a‒c, Fig. S3a‒c and Table S4 indicate the extent of dimethenamid-P, metazachlor, and pyroxasulfone sorption in the tested urban and agricultural soils. An increase in concentration of herbicides resulted in their increased sorption. Langmuir isotherm model revealed that an increase in initial herbicide concentration enhanced the sorption, and the maximum sorption capacities (Q_max_) for dimethenamid-P, metazachlor, and pyroxasulfone were in the range of 2.24 (QLD)–11.0 (CAL), 0.47 (QLD)–5.11 (CAL), and 4.18 (QLD)–28.56 (CAL) mg/kg, respectively (Fig. S3a‒c) and Table S4). Also, the ranges of Langmuir constants (*K*_L_) for dimethenamid-P, metazachlor, and pyroxasulfone were 0.96 (MAT)–1.17 (FLE), 0.74 (MAT)–1.32 (CAL), and 0.38 (FLE)–1.71 L/mg (MAT), respectively. Enhanced herbicide sorption in the soils could be related to the concentration gradient of the herbicides developed in aqueous phase of the soil (Agbaogun & Fischer, 2020). The herbicide sorption data were fitted in the Langmuir model with Langmuir coefficient values (*R*^2^, *P* < 0.05) of 0.866–0.985 for dimethenamid-P, 0.681–0.909 for metazachlor, and 0.499–0.985 for pyroxasulfone (Fig. S3a‒c and Table S4), indicating that herbicide sorption in urban and agricultural soils was multilayered onto heterogeneous surface sites but not limited to monolayer sorption on homogeneous surfaces.

Freundlich isotherm, on the other hand, is widely adopted for sorption that involves multilayers in energetically heterogeneous surfaces (Ozbay et al., 2018). In the present study, Freundlich model generated consistently higher *R*^2^ values (*P* < 0.05) in the range of 0.981–0.998, 0.898–0.998, and 0.871–0.999 for dimethenamid-P, metazachlor, and pyroxasulfone sorption, respectively (Fig. 4a‒c; Table S4). Again, the ranges of Freundlich constant (*K*_F_) values for dimethenamid-P, metazachlor, and pyroxasulfone were 16.42 (QLD)–42.03 (CAL), 12.14 (MAT)–44.09 (CAL), and 13.14 (TAR)–21.44 mg^1−1/*n*^ L^1/*n*^/g (CAL), respectively, indicating higher sorption capacity of the herbicides in the selected soils as observed earlier by Ozbay et al. (2018). Thus, the highest *K*_F_ value was observed particularly in soil CAL followed by soil FLE, probably due to large amounts of TOC (Fig. 4a‒c; Table S4). The lower *K*_F_ values in soil QLD and MAT confirm the mobility of herbicides in water sources from the soil surface. The 1/*n* values obtained for dimethenamid-P, metazachlor, and pyroxasulfone were in the range of 1.53 (CAL)–2.58 (QLD), 1.72 (MAT)–4.57 (QLD), and 1.18 (FLE)–2.39 (MAT), respectively, which is indicative of surface heterogeneity of the sorbent or sorption intensity, suggesting deviance from the linearity (Fig. 4a‒c; Table S4). Overall, 1/*n* < 1 suggests a favourable chemisorption mechanism involving heterogeneous surfaces, whereas 1/*n* > 1 denotes a cooperative sorption (Foo & Hameed, 2010). In the present study, 1/*n* > 1 indicates lesser heterogeneity of the sorbent surfaces (Jasper et al., 2020), clearly supporting the implication of undecomposed/partially-decomposed OM present in urban soils (Meftaul et al., 2020).

In all the soils, sorption of dimethenamid-P, metazachlor, and pyroxasulfone was significantly influenced by soil physicochemical properties. The initial fast sorption could be attributed to the partition of herbicides among clay surfaces and soil OM or partitioning into the rubbery segments of OM. The slow sorption observed here might be due to the gradual diffusion of herbicides into the soil micropores or typically cross-linked regions of OM or soil aggregates (Cheng et al., 2012). The surface sorption at lower equilibrium concentrations seems to control the overall herbicide sorption process. With increasing concentrations, sorption occur through the partition of herbicides when soil surface sorption sites are saturated. In the current study, herbicide sorption was directly proportional to soil OM and clay content (Ozbay et al., 2018). Of the soil minerals, quartz was the dominating constituent in the selected urban and agricultural soils and positively influenced the herbicide sorption. The soils with alkaline pH were less conducive to dimethenamid-P, metazachlor, and pyroxasulfone sorption than acidic soils (Okada et al., 2020; Padilla & Selim, 2020). Also, herbicide sorption positively correlated with the oxides of Al and Fe in soils (Agbaogun & Fischer, 2020). The alkyl (C–H) and carboxyl (C=O) groups of OM (Fig. 1; Table S2), associated with the polarity, cation-exchange, chemical reactivity and solubility, and wettability enhanced the herbicide sorption in the urban and agricultural soils. Overall, soil OM with acidic pH, carboxyl and alkyl groups, clay content, and Fe and Al oxides significantly influenced the herbicide sorption in urban and agricultural soils.

### Desorption of herbicides in the selected soils

**Fig. 5:**
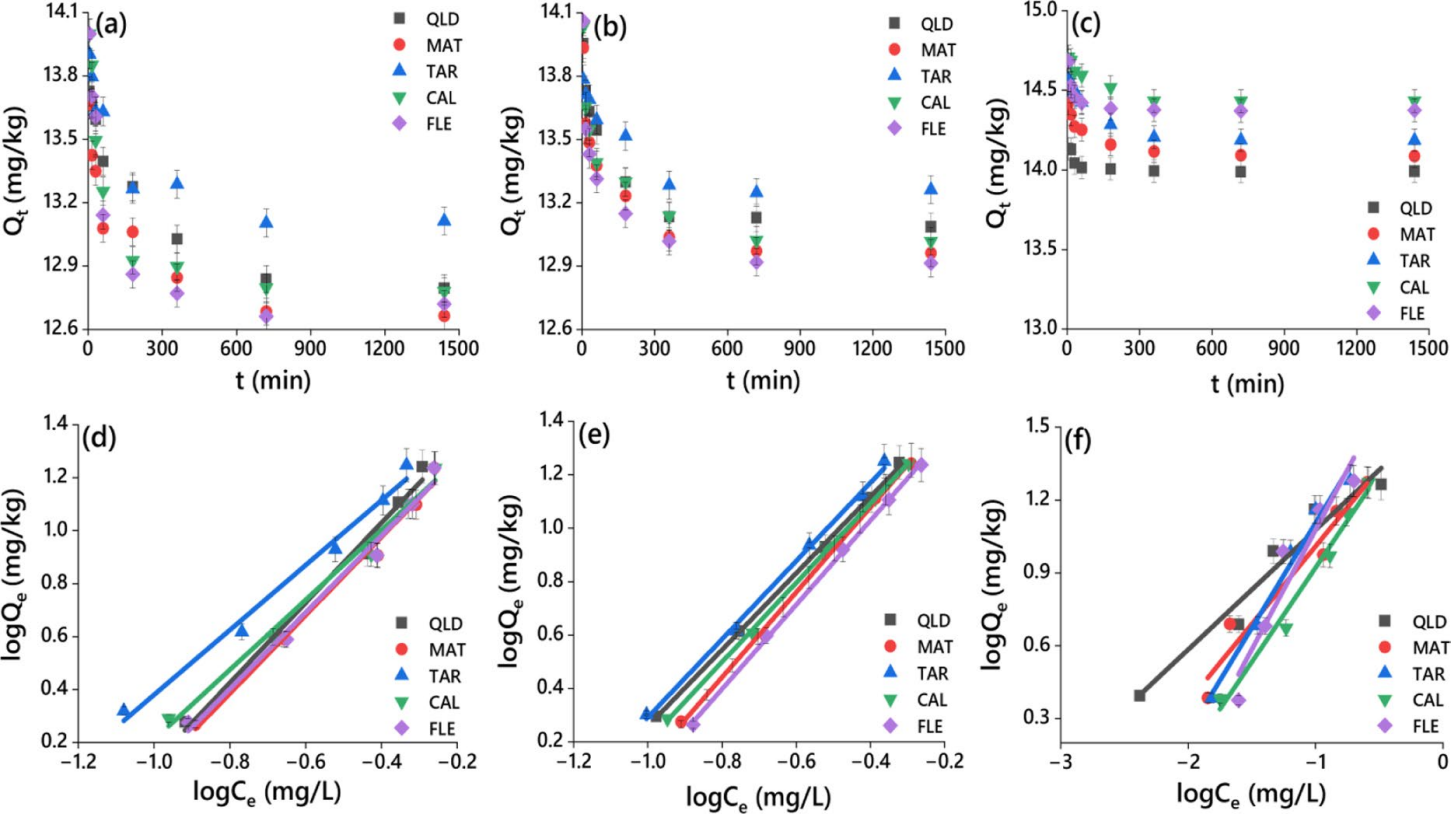
Equilibrium curves **a**, **b**, **c**, and Freundlich isotherms **d**, **e**, **f** of dimethenamid-P, metazachlor, and pyroxasulfone desorption in urban soils CAL and FLE, and agricultural soils QLD, MAT, and TAR. Q_t_ (mg/kg) is the amount of sorbed herbicide at time ‘t’; C_e_ (mg/L) is the equilibrium desorbed concentration and Q_e_ (mg/kg) is the equilibrium sorbed concentrations of the herbicide after one desorption cycle

Desorption is a process that determines the rate of discharge and potential distribution of herbicide in the soil matrix. The desorption process of the herbicides proceeded by an initial rapid release rate, followed by a gradual decline in the desorption process, and eventually reached an equilibrium at 24 h (Fig. 5a‒c). The *R*^2^ values (*P* < 0.05) of pseudo-first-order kinetics model of the five soils for dimethenamid-P, metazachlor, and pyroxasulfone were in the range of 0.310–0.539; 0.501–0.567, and 0.163–0.437, respectively (Fig. 5a‒c; Table S3) that were lower than those of the pseudo-second-order kinetics model. Hence, the desorption kinetics of the three herbicides in the selected soils were fitted well by a pseudo-second-order model with *R*^2^ values (*P* < 0.05) of 0.99–1.0 for dimethenamid-P, 0.99–1.0 for metazachlor, and 1.0 for pyroxasulfone. The amount of herbicide still sorbed, in terms of the equilibrium concentration after one desorption cycle, is expressed by desorption isotherms (Li et al., 2019). Thus, desorption data of three herbicides were best fitted with Langmuir and Freundlich models for the urban and farmland soils. The *R*^2^ values (*P* < 0.05) of the Langmuir isotherm in the tested soils for dimethenamid-P, metazachlor, and pyroxasulfone were in the range of 0.902–0.989, 0.874–0.952, and 0.012–0.977, respectively (Table S5), whereas the corresponding values of Freundlich isotherm were 0.986–0.989, 0.996–0.999, and 0.927–0.986, respectively (Fig. 5d‒f; Table S5). According to *R*^2^ values, the herbicide desorption data for five soils could be well described with the Freundlich model. Furthermore, *K*_F_ values for desorption of dimethenamid-P, metazachlor, and pyroxasulfone in the tested soils were in the range of 33.85–43.02, 45.74–57.0 and 37.30–131.1 mg^1−1/*n*^ L^1/*n*^/g, which were consistently greater than those obtained for sorption (16.42–42.03, 12.14–44.09 and 13.14–21.44 mg^1−1/*n*^ L^1/*n*^/g) (Fig. 5d‒f; Table S5). Thus, less desorption capacity of the herbicides was observed followed by the least portability into water sources from the surface of both urban and agricultural soils. Moreover, undecomposed/partially-decomposed OM, alkaline pH, and sand content in urban and agricultural soils enhanced herbicide desorption.

A delay or hinder in desorption process is assessed based on hysteresis (H) values (Li et al., 2023), wherein H values < 0.70 and 0.70‒1.0 are indicative of presence and absence of hysteresis, respectively (Martins et al., 2018). In fact, the H values of 0.70‒1.0 in soils indicate the irreversible sorption in clay minerals that can be ascribed to OM linked to soil aggregates as well as the entrapment of sorbed herbicides within meso- and micropores of both the OM and minerals (Li et al., 2023). The H values calculated were in the range of 0.51 (TAR)–0.88 (FLE), 0.31 (QLD)–0.92 (MAT), and 0.27 (MAT)–0.88 (FLE) for dimethenamid-P, metazachlor, and pyroxasulfone, respectively (Table 2). The H values of 0.70–1.0 in soils CAL and FLE for dimethenamid-P, MAR for metazachlor, and FLE for pyroxasulfone demonstrated no hysteresis process favouring desorption, whereas other soils with H values < 0.70 were in favour of hysteresis. Despite the fact that soils CAL and FLE contained relatively greater amounts of TOC than others, their leaching potentials were not significant as compared to other soils. This observation could be attributed to the presence of undecomposed/partially-decomposed OM in soils (Meftaul et al., 2020).

**Table 2:** Environmental and human health risks (mean values) of three herbicides in urban and agricultural soils

Soil ID	*K*_d_ (L/kg)	GUS	LIX	H	HQ_ing_ (Adults)	HQ_ing_ (Adolescent)	HQ_der_ (Adults)	HQ_der_ (Adolescent)	HQ_inh_ (Adults)	HQ_inh_ (Adolescent)
Dimethenamid−P										
QLD	9.12	0.38	< 0.02	0.58	1.58 × 10^−4^	1.43 × 10^−4^	8.21 × 10^−5^	1.04 × 10^−4^	2.03 × 10^−8^	1.84 × 10^−8^
MAT	9.54	0.50	< 0.02	0.61	1.60 × 10^−4^	1.45 × 10^−4^	8.32 × 10^−5^	1.05 × 10^−4^	2.06 × 10^−8^	1.86 × 10^−8^
TAR	11.24	1.51	< 0.02	0.51	1.68 × 10^−4^	1.51 × 10^−4^	8.71 × 10^−5^	1.10 × 10^−4^	2.16 × 10^−8^	1.95 × 10^−8^
CAL	23.16	1.82	< 0.02	0.85	1.94 × 10^−4^	1.75 × 10^−4^	1.00 × 10^−4^	1.28 × 10^−4^	2.50 × 10^−8^	2.26 × 10^−8^
FLE	20.52	0.96	< 0.02	0.88	1.90 × 10^−4^	1.72 × 10^−4^	9.89 × 10^−5^	1.25 × 10^−4^	2.45 × 10^−8^	2.22 × 10^−8^
Metazachlor										
QLD	7.80	0.57	< 0.02	0.31	2.25 × 10^−5^	2.04 × 10^−5^	1.17 × 10^−5^	1.48 × 10^−5^	5.75 × 10^−8^	2.62 × 10^−9^
MAT	9.32	0.63	< 0.02	0.92	2.37 × 10^−5^	2.14 × 10^−5^	1.23 × 10^−5^	1.56 × 10^−5^	6.11 × 10^−8^	2.76 × 10^−9^
TAR	8.12	2.06	0.02	0.34	2.28 × 10^−5^	2.06 × 10^−5^	1.18 × 10^−5^	1.50 × 10^−5^	5.88 × 10^−8^	2.66 × 10^−9^
CAL	16.92	2.44	0.11	0.69	2.76 × 10^−5^	2.50 × 10^−5^	1.43 × 10^−5^	1.82 × 10^−5^	7.12 × 10^−8^	3.22 × 10^−9^
FLE	12.99	1.47	< 0.02	0.67	2.61 × 10^−5^	2.36 × 10^−5^	1.35 × 10^−5^	1.72 × 10^−5^	6.72 × 10^−8^	3.04 × 10^−9^
Pyroxasulfone										
QLD	12.76	0.37	< 0.02	0.31	2.58 × 10^−4^	2.33 × 10^−4^	1.34 × 10^−4^	1.70 × 10^−4^	3.33 × 10^−8^	3.01 × 10^−8^
MAT	24.53	0.02	< 0.02	0.27	2.94 × 10^−4^	2.66 × 10^−4^	1.52 × 10^−4^	1.93 × 10^−4^	3.78 × 10^−8^	3.42 × 10^−8^
TAR	10.85	2.78	0.35	0.65	2.46 × 10^−4^	2.22 × 10^−4^	1.27 × 10^−4^	1.62 × 10^−4^	3.16 × 10^−8^	2.86 × 10^−8^
CAL	17.58	3.59	0.64	0.62	2.75 × 10^−4^	2.48 × 10^−4^	1.42 × 10^−4^	1.81 × 10^−4^	3.53 × 10^−8^	3.20 × 10^−8^
FLE	13.88	2.12	0.13	0.88	2.60 × 10^−4^	2.35 × 10^−4^	1.35 × 10^−4^	1.71 × 10^−4^	3.35 × 10^−8^	3.03 × 10^−8^

*K*_d_, Solid‒aqueous phase distribution coefficient; *GUS* Groundwater ubiquity score; *LIX* Leachability index; *H* Hysteresis index; HQ_ing_, Hazard quotient via ingestion; HQ_der_, Hazard quotient via dermal contact; HQ_inh_, Hazard quotient via inhalation; *QLD* Queensland; *MAT* Maitland; *TAR* Taree; *CAL* Callaghan; *FLE* Fletcher

### Principal component analysis

**Fig. 6:**
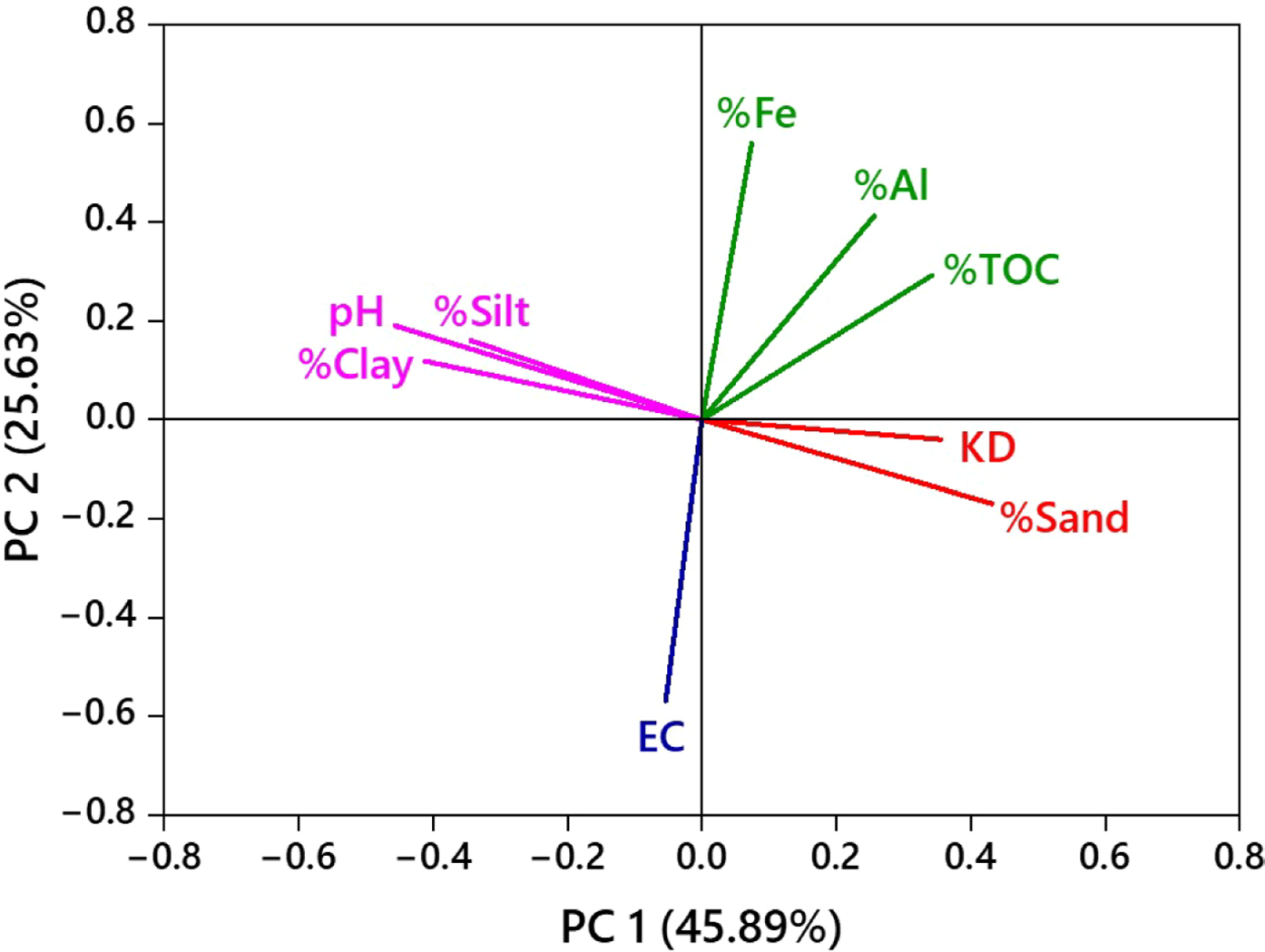
PCA score plots for dimethenamid-P, metazachlor, and pyroxasulfone sorption distribution coefficient (*K*_d_) in urban soils CAL and FLE, and agricultural soils QLD, MAT, and TAR

Principal component analysis (PCA) was performed to explore the mutual interactions among multiple soil parameters and *K*_d_ of the herbicides, and the data are presented in Fig. 6, Tables S6 and S7. Among all the soil parameters, OM (*R*^2^ = 0.47, *P* < 0.05), Al oxides (*R*^2^ = 0.33, *P* < 0.05), and contents of sand (*R*^2^ = 0.56, *P* < 0.05) positively correlated with *K*_d_ values of the herbicides (*R*^*2*^ = 1.0, *P* < 0.05), with a variance of 25.63, 16.90 and 3.85%, respectively (Fig. 6; Table S6). The eigenvalues associated with the above parameters were 2.30, 1.52 and 0.34, respectively (Fig. 6; Table S7). First two principal components contributed to nearly 71.52% of the cumulative variance and an eigenvalue of 4.13. On the other hand, contents of clay (*R*^2^ = ‒ 0.43, *P* < 0.05), silt (*R*^2^ = ‒ 0.51, *P* < 0.05), Fe oxides (*R*^2^ = ‒ 0.02, P < 0.05), alkaline pH (*R*^2^ = ‒ 0.57, *P* < 0.05), and EC (*R*^2^ = ‒ 0.03, *P* < 0.05) showed a strong negative correlation with herbicide *K*_d_ values (*R*^*2*^ = 1.000, *P* < 0.05) (Fig. 6; Table S6).

Generally, OM in soil enhanced sorption of the herbicides because of the presence of C=O, O–H, C–H, C=C groups through covalent/electrostatic/H-bonds (Fig. 1). Among the four, C=O and C–H groups were more favourable as they are associated with solubility, cation-exchange, polarity and chemical reactivity, and wettability (Capriel et al., 1995; Celi et al., 1997; Heller et al., 2015). Herbicide sorption in soil strongly depends on both the quantity and quality of OM; however, well-decomposed OM sorbed significantly greater amounts of herbicide than undecomposed/partially-decomposed OM (Ozbay et al., 2018). Likewise, negatively charged clay minerals react with herbicides via cation-exchange or electrostatic interactions. Also, soil clay minerals might immobilize the herbicides upon their contact because the metal ions form surface complexes (Wu et al., 2021). Mostly, herbicides with negative charges have a high affinity to oxides of Fe and Al in clay fraction and sorbed strongly via cation-exchange in the soil matrix (Okada et al., 2020). Owing to electrostatic repulsion that results from more net negative surface charges of soil minerals, sorption of herbicides is inversely proportional to soil alkaline pH, and vice versa (Okada et al., 2020)*.* In fact, electrostatic repulsion increases in herbicides with more negative net charges due to deprotonation at alkaline pH (Padilla & Selim, 2020). Thus, PCA confirms that the contents of clay, silt and Fe, and alkaline pH had a negative correlation with herbicide *K*_d_, whereas a positive correlation was observed with soil OM, Al oxides, content of sand and acidic pH.

### Human non-carcinogenic health hazards

**Fig. 7:**
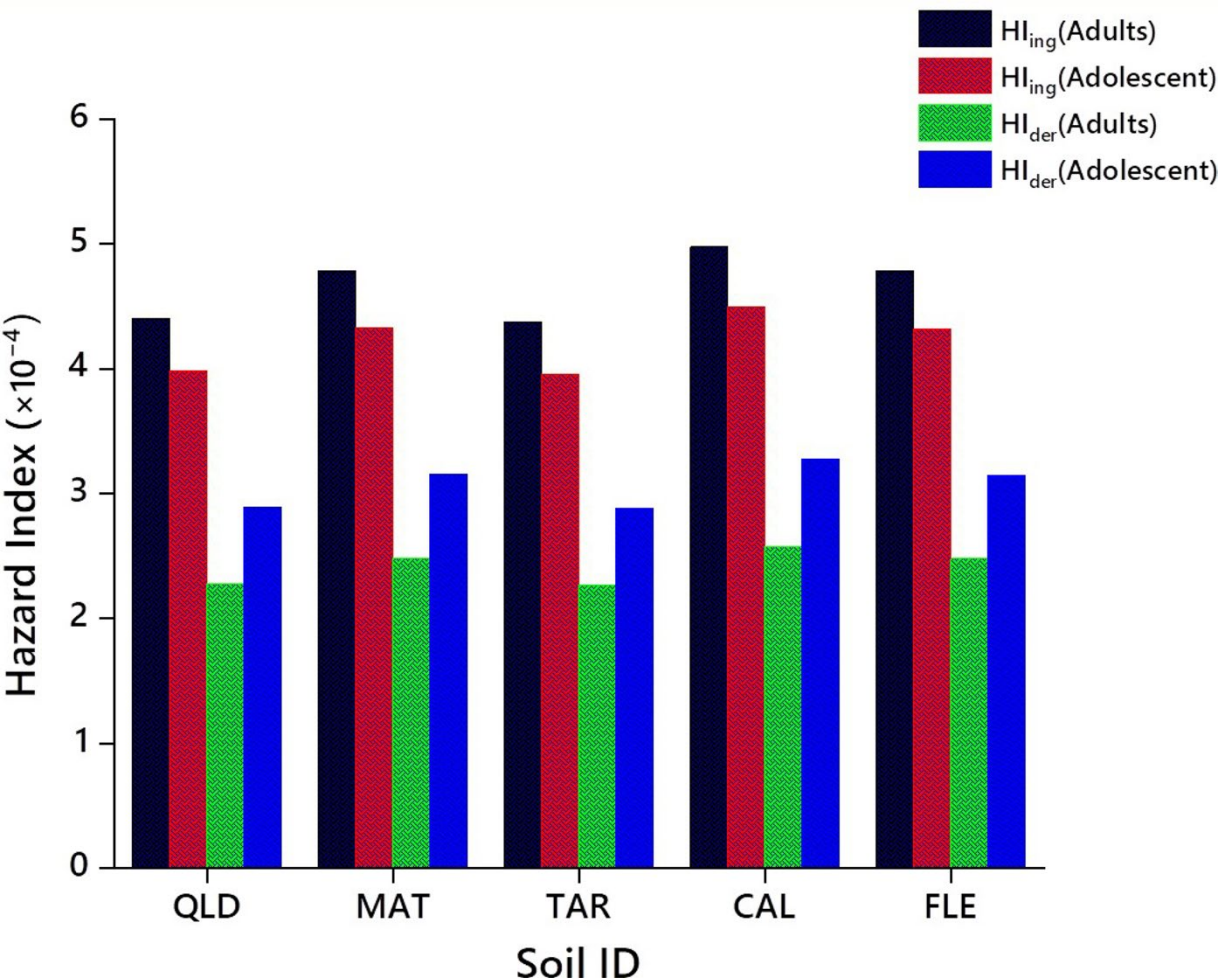
Potential non-cancer health risk in terms of hazard index (HI) for human adults and adolescents exposed to urban soils CAL and FLE, and agricultural soils QLD, MAT, and TAR contaminated with dimethenamid-P, metazachlor, and pyroxasulfone

Human carcinogenic and non-cancer risks associated with the herbicide exposure were evaluated using widely-adopted equations and constant parameters available in the literarure (Table 1), and the data obtained in the present study for adults and adolescents are shown in Table 2 and Fig. 7. The non-dietary CDI values that resulted from exposure to urban and agricultural soils contaminated with dimethenamid-P, metazachlor, and pyroxasulfone through pathways of ingestion, dermal and inhalation for human adults were in the range of 5.53–5.88 × 10^–6^, 2.34–3.05 × 10^–6^ and 5.80–7.57 × 10^–10^, and 4.08–5.32 × 10^−6^; 2.97–3.87 × 10^–6^ and 5.25–6.84 × 10^−10^ mg/kg/day for adolescents, respectively (data not shown). The non-cancer risk of herbicides for a given exposure pathway is expressed as the hazard quotient (HQ). This quotient represents the ratio of the average non-dietary CDI assessed to the reference dose (mg/kg/day) of the herbicide. The ranges in mean values of HQ for dimethenamid-P, metazachlor, and pyroxasulfone in urban and agricultural soils via pathways of ingestion, dermal and inhalation pathways in human adults were 2.25 × 10^−5^–2.94 × 10^−4^, 1.17 × 10^−5^–1.52 × 10^−4^ and 3.78 × 10^−8^–7.12 × 10^−8^, and the corresponding values in adolescents 2.04 × 10^−5^–2.66 × 10^−4^, 1.48 × 10^−5^–1.93 × 10^−4^ and 2.62 × 10^−9^–3.42 × 10^−8^, respectively (Table 2). The HQ values of three herbicides for urban and agricultural soils were significantly lower than the recommended threshold limit of < 1.00 (Bhandari et al., 2020), which indicates lack of considerable non-cancer risks for adolescents and adults. Also, the mean HI values of dimethenamid-P, metazachlor, and pyroxasulfone through the above-mentioned pathways for soils QLD, MAT, TAR, CAL and FLE were 1.11 × 10^−4^–4.39 × 10^−4^, 3.41 × 10^−8^–4.78 × 10^−4^, 7.93 × 10^−8^–4.37 × 10^−4^, 1.29 × 10^−7^–4.97 × 10^−4^ and 1.81 × 10^−7^–4.77 × 10^−4^ (adults), and 5.11 × 10^−8^–3.97 × 10^−4^, 5.56 × 10^−8^–4.32 × 10^−4^, 5.08 × 10^−8^–3.95 × 10^−4^, 5.78 × 10^−8^–4.49 × 10^−4^ and 5.55 × 10^−8^–4.31 × 10^−4^ (adolescent), respectively (Fig. 7). The HI value of herbicides for urban and agricultural soils was also significantly lesser than the threshold value of < 1.0 (Bhandari et al., 2020), demonstrating no considerable non-cancer risks for adults and adolescents. In all, the current study reveals that exposure to the selected herbicide-contaminated urban and farmland soils through pathways of ingestion, dermal and inhalation causes negligible or no non-carcinogenic risk to human adults and adolescents.

### Environmental risks of the selected herbicides

The pollution of surface and groundwater with herbicides is a rising concern all over the world due to their toxicity toward nontarget biota, decline in potable water quality as well as food safety. The evaluation of environmental hazards of herbicides through leaching potential was done by calculating *K*_oc_ using *K*_d_ (Martins et al., 2018). Using half-lives (t½, days) of the herbicides and *K*_oc_, the values of environmental health indices such as GUS, and LIX were determined, and the data obtained are presented in Table 2. The t½ of 16, 30 and 156 days for dimethenamid-P, metazachlor, and pyroxasulfone, respectively, were considered here although these values might vary based on environmental conditions like microbial activities, temperature and soil depth (Tudi et al., 2021). The ranges in *K*_d_ values obtained for dimethenamid-P, metazachlor, and pyroxasulfone in the selected urban and agricultural soils were 9.12–23.16, 7.80–16.92, and 10.85–17.58 L kg^−1^, respectively, indicating the favourable sorption followed by limited portability of the herbicides (Table 2).

The calculated LIX values for dimethenamid-P, metazachlor, and pyroxasulfone were < 0.02 (for all soils), < 0.02 (soils QLD, MAT, and FLE)–0.11 (soil CAL), and < 0.02 (soils QLD and MAT)–0.64 (soil CAL) (Table 2). Overall, the LIX values varied between minimum (0.0) and maximum (1.0) leaching potentials (Martins et al., 2018). Thus, the calculated LIX values for the three herbicides in the urban and agricultural soils indicated moderate (soils CAL and TAR for pyroxasulfone) to least (soils QLD, MAT, and FLE) leaching potential, indicating their potential in contaminating watercourses. In contrast, the GUS values calculated were in the range of 0.38 (soil QLD)–1.82 (soil CAL), 0.57 (soil QLD)–2.44 (soil CAL), and 0.02 (soil QLD)–3.59 (soil CAL) for dimethenamid-P, metazachlor, and pyroxasulfone, respectively (Table 2). Generally, soils with GUS values of > 2.80, 1.80–2.80, and < 1.80 are considered ‘leachers’, ‘transitional’ and ‘non-leachers’, respectively (Martins et al., 2018). Thus, only soil CAL is the potential leacher for pyroxasulfone, while soils FLE and TAR, soils CAL and TAR, and soil CAL were transitional for pyroxasulfone, dimethenamid-P, and metazachlor, respectively. The GUS, and LIX values indicated the moderate to least portability of herbicides into watercourses from soil surface thereby causing potential environmental health hazards. Overall, the present investigation comprehensively assessed the health risks in humans and environment due to soil contamination of herbicides, dimethenamid-P, metazachlor, and pyroxasulfone, that are widely used globally for plant protection.

## Conclusion

The present study investigated the distribution, fate, and exposure risks of three widely used effective pre-emergence herbicides, viz., dimethenamid-P, metazachlor, and pyroxasulfone in well-characterized two urban (CAL and FLE) and three agricultural soils (QLD, MAT, and TAR) following batch experiments. The kinetics of herbicide sorption and desorption were perfectly described by the pseudo-second-order model, while the isotherm data aligned well with the Freundlich model. The factors that played a pivotal role in influencing the sorption and desorption processes of the selceted herbicides in five soils include decomposed OM rich in C–H and C=O groups, contents of clay and silt, Al and Fe oxides, and slightly acidic pH. It is noteworthy that the urban soils exhibited slightly higher sorption capacities as compared to the agricultural soils, and followed the order: QLD < MAT < TAR < FLE < CAL. Although urban soils contained significantly greater amounts of OM than agricultural soils, the OM in urban soils was mostly in undecomposed/partially decomposed form. Thus, the minor differences observed in the overall sorption processes could be ascribed to the nature of OM in soils. PCA revealed a negative correlation between *K*_d_ of the herbicides and soil characteristics such as Fe oxides, alkaline pH, clay and silt content in soils, while a positive correlation was evident with TOC, Al and sand content. The claculated HQ and HI values indicated that human exposure to soils contaminated with the selected herbicides through dermal, ingestion, and inhalation pathways result in either negligible or no non-carcinogenic risks for both adults and adolescents. Furthermore, H, GUS, and LIX values demonstrated that the mobility of herbicides from urban and agricultural soils into watercourses is generally moderate to low, indicating their impact toward nontarget biota and food safety. Our findings thus assist farmers in the judicious application of herbicides in controlling weeds in farmlands, and the regulatory authorities in protecting human and environmental health.

### Supplementary Information

Below is the link to the electronic supplementary material.

Supplementary file1 (DOCX 189 kb)

## Data Availability

The datasets used in this study are available from the corresponding author on reasonable request.
