# Supplementary material for: Pre-emergence herbicides widely used in urban and farmland soils: fate, and potential human and environmental health risks
Source: Environ Geochem Health. 2024 Mar 14;46(4):132. doi: 10.1007/s10653-024-01907-6 (PMC10940459; doi:10.1007/s10653-024-01907-6)
Supplement: Supplementary file 1 — Supplementary file1 (DOCX 189 kb) [file 10653_2024_1907_MOESM1_ESM.docx]

***Supplementary Information***

**Pre-emergence herbicides widely used in urban and farmland soils: fate, and potential human and environmental health risks**

**Aney Parven** **• Islam Md Meftaul • Kadiyala Venkateswarlu • Saianand Gopalan • Mallavarapu Megharaj**^*^

**A. Parven • I. M. Meftaul • S. Gopalan • M. Megharaj**

Global Centre for Environmental Remediation (GCER), College of Engineering, Science and Environment, The University of Newcastle, Callaghan, NSW 2308, Australia

**A. Parven • I. M. Meftaul**

Department of Agricultural Chemistry, Sher-e-Bangla Agricultural University, Dhaka-1207, Bangladesh

**K. Venkateswarlu**

Formerly Department of Microbiology, Sri Krishnadevaraya University, Anantapuramu 515003, India

**M. Megharaj**

crc for Contamination Assessment and Remediation of the Environment (crcCARE), ATC Building, University Drive, Callaghan, NSW 2308

****Address for correspondence***:

**Prof. Mallavarapu Megharaj**

Global Centre for Environmental Remediation (GCER)

College of Engineering, Science and Environment

The University of Newcastle, ATC Building

University Drive, Callaghan, NSW 2308, Australia

Mobile: +61 411126857; orcid.org/0000-0002-6230-518X

E-mail: [megh.mallavarapu@newcastle.edu.au](mailto:megh.mallavarapu@newcastle.edu.au)


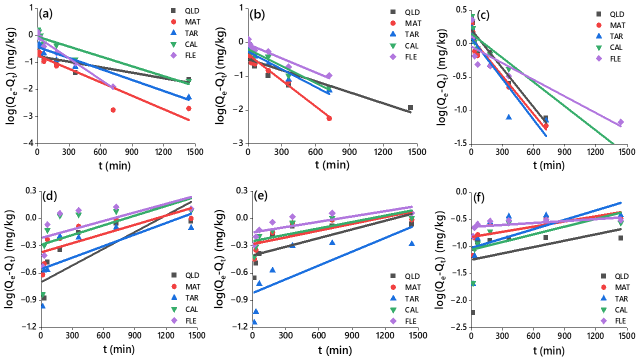


**Fig. S1** Pseudo-first-order kinetics for sorption (**a, b, c**) and desorption (**d, e, f**) of dimethenamid-P, metazachlor, and pyroxasulfone in five urban and agricultural soils. Q_t_ (mg/kg) is the amount of sorbed herbicide at time ‘t’ and Q_e_ (mg/kg) are the equilibrium sorbed concentration of herbicide, respectively.


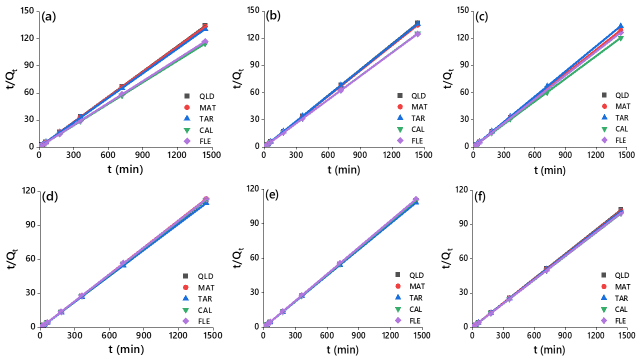


**Fig. S2** Pseudo-second-order kinetics for sorption (**a, b, c**) and desorption (**d, e, f**) of dimethenamid-P, metazachlor, and pyroxasulfone in five urban and agricultural soils. Q_t_ (mg/kg) is the amount of sorbed herbicide at time ‘t’.

**
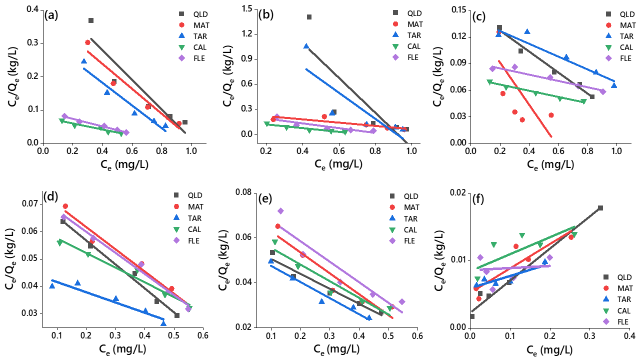
**

**Fig. S3** Langmuir isotherms for sorption (**a, b, c**) and desorption (**d, e, f**) of dimethenamid-P, metazachlor, and pyroxasulfone in five urban and agricultural soils. C_e_ (mg/L) and Q_e_ (mg/kg) are the equilibrium concentration and equilibrium sorbed concentration of herbicide, respectively.

**Table S1** Physicochemical properties of the selected urban and agricultural soils

| Soil type | Soil ID |  | pH | EC | TOC | Al (%) | Fe (%) | Sand (%) | Silt (%) | Clay (%) | Texture | Major minerals |
| --- | --- | --- | --- | --- | --- | --- | --- | --- | --- | --- | --- | --- |
| Agricultural | QLD |  | 9.15 | 125.16 | 0.19±0.21 | 0.81±0.14 | 1.08±0.06 | 28.80 | 41.20 | 30.0 | Loam | Quartz, Spinel, Magnetite, Franklinite |
|  | MAT |  | 6.78 | 124.50 | 0.25±0.01 | 0.65±0.05 | 1.42±0.15 | 76.30 | 16.20 | 7.50 | Loamy sand | Quartz, Albite, Oligoclash, Hydrosodalite, |
|  | TAR |  | 7.51 | 161.46 | 2.02±0.01 | 1.19±0.03 | 0.92±0.11 | 33.8 | 55 | 11.20 | Silt loam | Quartz, Albite, Sinnerite, Bernalite |
| Urban | CAL |  | 6.2 | 123.36 | 7.66±0.01 | 1.95±0.05 | 1.27±0.13 | 68.8 | 23.80 | 7.40 | Sandy loam | Quartz, Marshite, Albite, Zeolite |
|  | FLE |  | 6.6 | 190.46 | 1.29±0.02 | 0.03±0.67 | 0.01±0.99 | 63.8 | 23.80 | 12.40 | Sandy loam | Quartz, Oligoclase, Albite, Sodalite |

**Table S2** FTIR spectral characteristics of the selected urban and agricultural soils

| Components | Vibrations | Wavelength range (cm) | References |
| --- | --- | --- | --- |
| Aromatics | C–H in plane | 1025–1200 | Janik et al. (2007) |
| Alkenes group amides, COO−/aromatics/O–H stretching | C=C stretching | 1680–1640 | Changwen et al. (2007); Calderón et al. (2013) |
| Alkanes group, aliphatic methyl, and methylene groups | C=O stretching | 1800–1950 | Xing et al. (2019) |
| Alkanes group, aliphatic methyl, and methylene groups | C–H stretching | 2800–2950 | Soriano-Disla et al. (2014) |
| Carboxyl, alcohols, phenols/amine, and amide groups | O–H stretching, H-bonded | 3200–3450 | Bernier et al. (2013) |
| Hydroxyl groups | O–H stretching, ‘free’ hydroxyl | 3600–3750 | Xing et al. (2019) |

**Table S3** Constants and coefficients used for the determination of pseudo-first-order and pseudo-second-order kinetics models of dimethenamid-P, metazachlor, and pyroxasulfone sorption and desorption in urban and agricultural soils

| Soil ID | Pseudo-first-order kinetics model | | | | Pseudo-second-order kinetics model | | | |
| --- | --- | --- | --- | --- | --- | --- | --- | --- |
|  | *K*_1(Sor)_ | *K*_1(Des)_ | *R*^2^_(Sor)_ | *R*^2^_(Des)_ | *K*_2(Sor)_ | *K*_2(Des)_ | *R*^2^_(Sor)_ | *R*^2^_(Des)_ |
| Dimethenamid-P | | | | | | | | |
| QLD | 6.77×10^‒4^ | 6.13×10^‒4^ | 0.688 | 0.493 | 0.20 | 0.02 | 1.000 | 0.999 |
| MAT | 1.66×10^‒3^ | 3.37×10^‒4^ | 0.822 | 0.539 | 0.17 | 0.03 | 1.000 | 0.999 |
| TAR | 1.35×10^‒3^ | 4.23×10^‒4^ | 0.913 | 0.459 | 0.07 | 0.04 | 1.000 | 0.999 |
| CAL | 1.21×10^‒3^ | 3.55×10^‒4^ | 0.873 | 0.310 | 0.02 | 0.04 | 0.999 | 1.000 |
| FLE | 2.62×10^‒3^ | 3.13×10^‒4^ | 0.970 | 0.371 | 0.03 | 0.05 | 1.000 | 0.999 |
| Metazachlor | | | | | | | | |
| QLD | 1.09×10^‒3^ | 3.17×10^‒4^ | 0.805 | 0.501 | 0.09 | 0.05 | 1.000 | 1.000 |
| MAT | 2.57×10^‒3^ | 2.47×10^‒4^ | 0.957 | 0.567 | 0.07 | 0.04 | 1.000 | 1.000 |
| TAR | 1.75×10^‒3^ | 5.05×10^‒4^ | 0.949 | 0.527 | 0.04 | 0.06 | 0.999 | 1.000 |
| CAL | 1.67×10^‒3^ | 2.33×10^‒4^ | 0.936 | 0.560 | 0.03 | 0.04 | 0.999 | 0.999 |
| FLE | 1.39×10^‒3^ | 1.94×10^‒4^ | 0.917 | 0.559 | 0.02 | 0.04 | 0.999 | 1.000 |
| Pyroxasulfone | | | | | | | | |
| QLD | 2.01×10^‒3^ | 3.92×10^‒4^ | 0.918 | 0.163 | 0.01 | 0.69 | 0.999 | 1.000 |
| MAT | 1.98×10^‒3^ | 3.18×10^‒4^ | 0.915 | 0.437 | 0.02 | 0.13 | 0.999 | 1.000 |
| TAR | 2.10×10^‒3^ | 5.77×10^‒4^ | 0.856 | 0.387 | 0.02 | 0.10 | 0.999 | 1.000 |
| CAL | 2.43×10^‒3^ | 4.74×10^‒4^ | 0.939 | 0.369 | 0.01 | 0.16 | 0.999 | 1.000 |
| FLE | 1.81×10^‒3^ | 1.19×10^‒4^ | 0.822 | 0.321 | 0.02 | 0.48 | 0.999 | 1.000 |

*K*_1_ and *K*_2_ are the dimensionless parameters describing the rate constant of pseudo-first-order and pseudo-second-order kinetic, respectively; *R*^2^, Coefficient of determination.

**Table S4** Isotherm parameters for dimethenamid-P, metazachlor, and pyroxasulfone sorption in urban and agricultural soils

| Soil ID | Langmuir isotherm | | | Freundlich isotherm | | |
| --- | --- | --- | --- | --- | --- | --- |
|  | *Q*_max_  (mg/kg) | *K*_L_  (L/mg) | *R*^2^ | *K*_F_  (mg^1-1/^*^n^* L^1/^*^n^*/g) | 1*/n* | *R*^2^ |
| Dimethanamid-P | | | | | | |
| QLD | 2.24 | 0.97 | 0.866 | 16.42 | 2.58 | 0.998 |
| MAT | 2.66 | 0.96 | 0.944 | 16.74 | 2.40 | 0.992 |
| TAR | 2.92 | 1.07 | 0.944 | 23.09 | 2.39 | 0.995 |
| CAL | 11.00 | 1.16 | 0.923 | 42.03 | 1.53 | 0.981 |
| FLE | 8.88 | 1.17 | 0.985 | 38.84 | 1.62 | 0.987 |
| Metazachlor | | | | | | |
| QLD | 0.47 | 1.07 | 0.681 | 17.70 | 4.57 | 0.973 |
| MAT | 5.11 | 0.74 | 0.743 | 12.14 | 1.72 | 0.898 |
| TAR | 0.64 | 1.08 | 0.712 | 18.72 | 4.21 | 0.981 |
| CAL | 4.47 | 1.32 | 0.909 | 44.09 | 2.14 | 0.998 |
| FLE | 3.49 | 1.13 | 0.802 | 29.91 | 2.32 | 0.994 |
| Pyroxasulfone | | | | | | |
| QLD | 8.42 | 0.78 | 0.985 | 18.62 | 1.56 | 0.994 |
| MAT | 4.18 | 1.71 | 0.499 | 18.05 | 2.39 | 0.871 |
| TAR | 13.80 | 0.51 | 0.924 | 13.14 | 1.32 | 0.982 |
| CAL | 28.57 | 0.47 | 0.981 | 21.44 | 1.20 | 0.999 |
| FLE | 28.11 | 0.38 | 0.934 | 16.07 | 1.18 | 0.993 |

*Q*_max_, Sorption capacity; *K*_L_, Langmuir constant; *R*^2^, Coefficient of determination; *K*_F_, Freundlich constant; 1*/n*, Sorption intensity.

**Table S5** Isotherm parameters for dimethenamid-P, metazachlor, and pyroxasulfone desorption in urban and agricultural soils

| Soil ID | Langmuir isotherm | | Freundlich isotherm | | |
| --- | --- | --- | --- | --- | --- |
|  | *K*_L_  (L/mg) | *R*^2^ | *K*_F_  (mg^1-1/^*^n^* L^1/^*^n^*/g) | 1*/n* | *R*^2^ |
| Dimethenamid-P | | | | | |
| QLD | 1.17 | 0.989 | 43.02 | 1.51 | 0.986 |
| MAT | 1.04 | 0.972 | 36.82 | 1.47 | 0.989 |
| TAR | 0.82 | 0.902 | 40.19 | 1.22 | 0.986 |
| CAL | 0.85 | 0.965 | 33.85 | 1.31 | 0.988 |
| FLE | 1.02 | 0.988 | 36.27 | 1.44 | 0.986 |
|  |  |  | Metazachlor |  |  |
| QLD | 1.16 | 0.947 | 49.11 | 1.43 | 0.999 |
| MAT | 1.28 | 0.898 | 51.99 | 1.59 | 0.997 |
| TAR | 1.30 | 0.952 | 57.00 | 1.46 | 0.996 |
| CAL | 1.17 | 0.917 | 48.42 | 1.48 | 0.999 |
| FLE | 1.18 | 0.874 | 45.74 | 1.57 | 0.999 |
|  |  |  | Pyroxasulfone |  |  |
| QLD | 20.82 | 0.977 | 37.30 | 0.49 | 0.963 |
| MAT | 6.88 | 0.842 | 47.25 | 0.66 | 0.958 |
| TAR | 3.05 | 0.809 | 95.21 | 0.87 | 0.986 |
| CAL | 2.98 | 0.638 | 48.20 | 0.75 | 0.984 |
| FLE | 0.36 | 0.012 | 131.11 | 1.04 | 0.927 |

*Q*_max_, Sorption capacity; *K*_L_, Langmuir constant; *R*^2^, Coefficient of determination; *K*_F_, Freundlich constant; 1*/n*, Sorption intensity.

**Table S6** Relationship, in terms of correlation matrix, between soil properties, and distribution coefficient (*K*_d_) values of dimethenamid-P, metazachlor, and pyroxasulfone in urban and agricultural soils

|  | *K*_d_ | TOC | %Al | %Fe | %Sand | %Silt | %Clay | pH | EC |
| --- | --- | --- | --- | --- | --- | --- | --- | --- | --- |
| *K*_d_ | 1.0 | 0.47804 | 0.33153 | ‒0.01703 | 0.56751 | ‒0.51821 | ‒0.43047 | ‒0.5714 | ‒0.03383 |
| TOC | 0.47804 | 1.0 | 0.95924 | 0.20897 | 0.30905 | ‒0.14867 | ‒0.45931 | ‒0.56 | ‒0.22139 |
| %Al | 0.33153 | 0.95924 | 1.0 | 0.34941 | 0.08274 | 0.08898 | ‒0.33987 | ‒0.36567 | ‒0.32714 |
| %Fe | ‒0.01703 | 0.20897 | 0.34941 | 1.0 | 0.0703 | ‒0.05253 | ‒0.07296 | 0.11379 | ‒0.93688 |
| %Sand | 0.56751 | 0.30905 | 0.08274 | 0.0703 | 1.0 | ‒0.91918 | ‒0.74834 | ‒0.85262 | ‒0.06345 |
| %Silt | ‒0.51821 | ‒0.14867 | 0.08898 | ‒0.05253 | ‒0.91918 | 1.0 | 0.42662 | 0.60937 | 0.17082 |
| %Clay | ‒0.43047 | ‒0.45931 | ‒0.33987 | ‒0.07296 | ‒0.74834 | 0.42662 | 1.0 | 0.93168 | ‒0.14198 |
| pH | ‒0.5714 | ‒0.56 | ‒0.36567 | 0.11379 | ‒0.85262 | 0.60937 | 0.93168 | 1.0 | ‒0.21846 |
| EC | ‒0.03383 | ‒0.22139 | ‒0.32714 | ‒0.93688 | ‒0.06345 | 0.17082 | ‒0.14198 | ‒0.21846 | 1.0 |

**Table S7** Eigenvalues of the correlation matrix

| S. No. | Eigenvalue | Variance (%) | Cumulative % |
| --- | --- | --- | --- |
| 1 | 4.13012 | 45.89 | 45.89 |
| 2 | 2.3068 | 25.63 | 71.52 |
| 3 | 1.52134 | 16.90 | 88.43 |
| 4 | 0.69484 | 7.72 | 96.15 |
| 5 | 0.3469 | 3.85 | 100 |
| 6 | 0 | 0 | 100 |
| 7 | 0 | 0 | 100 |
| 8 | 0 | 0 | 100 |
| 9 | 0 | 0 | 100 |

**References**

Bernier, M. -H., Levy, G. J., Fine, P., & Borisover, M. (2013). Organic matter composition in soils irrigated with treated wastewater: FTIR spectroscopic analysis of bulk soil samples. *Geoderma*, *209*, 233–240. https://doi.org/10.1016/j.geoderma.2013.06.017

Calderón, F., Haddix, M., Conant, R., Magrini-Bair, K., & Paul, E. (2013). Diffuse-reflectance Fourier-transform mid-infrared spectroscopy as a method of characterizing changes in soil organic matter. *Soil Science Society of America Journal*, *77*, 1591–1600. https://doi.org/10.2136/sssaj2013.04.0131

Changwen, D., Linker, R., & Shaviv, A. (2007). Characterization of soils using photoacoustic mid-infrared spectroscopy. *Applied Spectroscopy*, *61*, 1063–1067. https://opg.optica.org/as/abstract.cfm?URI=as-61-10-1063

Janik, L. J., Skjemstad, J., Shepherd, K., & Spouncer, L. (2007). The prediction of soil carbon fractions using mid-infrared-partial least square analysis. *Soil Research*, *45*, 73–81. https://doi.org/10.1071/SR06083

Soriano-Disla, J. M., Janik, L. J., Viscarra Rossel, R. A., Macdonald L. M., & McLaughlin, M. J. (2014). The performance of visible, near-, and mid-infrared reflectance spectroscopy for prediction of soil physical, chemical, and biological properties. *Applied Spectroscopy Reviews*, *49*, 139–186. https://doi.org/10.1080/05704928.2013.811081

Xing, Z., Tian, K., Du, C., Li, C., Zhou, J., & Chen, Z. (2019). Agricultural soil characterization by FTIR spectroscopy at micrometer scales: depth profiling by photoacoustic spectroscopy. *Geoderma*, *335*, 94–103. https://doi.org/10.1016/j.geoderma.2018.08.003
